# Department of Surgery

**Published:** 1893-11

**Authors:** 

**Affiliations:** Galveston


### Current Medical Literature.

### DEPARTMENT OF SURGERY.

[Edited by Prof. J. E. Thompson, M. D., Galveston.]

### Abstracts From Late Surgical Journals.

NOLEN.—A new method of treatment in exudative tubercular peritonitis.—(Berliner Klin. Wochenschrift, 1893, No. 3.)

The author, on investigating the reasons for the improvement noticed in patients whose abdominal cavities had been washed out, came to the conclusion that the change was brought about by the contact of the diseased parts with air. In three cases, after having previously removed the ascitic fluid, he pumped air into the peritoneal cavity. By pressure and kneading, the air was brought into contact with all the abdominal viscera. The air was removed in about five minutes from the time of introduction.

The ascites permanently remained absent in all three cases after insufflation. In two cases, which unfortunately were not long under observation, cure resulted.

RAMANGÉ—"Enteroplexy."—Jacobo Teuser, Buenos Ayres, 1893.

Enteroplexy is a name given by R. to a proceeding he has devised for uniting the severed ends of intestine without sutures. The instrument used is somewhat similar to Murphy's button, but larger. It is a kind of a clamp consisting of two smooth aluminum rings, which are united together by two pegs on one ring fitting into the slots of the other. They are placed in the severed ends of the intestine which are united over them and secured by a few sutures. Then the rings, having between them the double thickness of the infolded intestine, are pressed together and fixed by the pegs. The instrument is subsequently shed into the bowel and voided per anum.

Experimentation on pigs proved the efficacy of the instruments.

---

O. WYES—On the treatment of hydrocephalus. (Korrespondenzblatt für Schueizer Arzte, 1893, No. 8.)

Out of 43 patients affected with hydrocephalus, presenting themselves at the children's hospital in Zurich, 31 died, 10 lived, and two were not followed. The ages of the surviving patients varied between 3 and 21. Twenty-four cases were congenital and 16 were acquired, the former showing naturally a worse prognosis and a less favorable result.

The earlier cases were treated by counter-irritation, iron preparation, malt extract and baths. The later cases were treated some by puncture of the cranial bones, others by puncture of the subdural space in the lumbar region of the spinal column. In one case, a 7½ months child in whom intracranial pressure had caused blindness, puncture was performed seven times, five times in the neighborhood of the anterior fontanelle, twice in the lumbar region. He claims that removal of hydrocephalic fluid produces a marked improvement in patients. Convulsions cease, excitation is lessened, bodily development goes on apace, power of speech returns and sight is restored.

---

QUÉNCE.—(Compte Rendu du Congres de Chirugie—Revue de Chirugie. No. 5, 1893, p. 405.)

Operative treatment of hæmorrhoids by a new procedure.

The author modifies Whitehead's operation by separating the mucous membrane from its bed only opposite the pile masses, leaving it attached at the muco-cutaneous margins in the intervals. After separation he excises the masses without the slightest loss of blood, and unites the raw edges by a few points of suture. He packs the rectum with iodoform gauze. He claims for this procedure that there is no traction on the line of suture and that the wound is not likely to become infected; also that the operation can be done even when the piles are inflamed.

---

ZANCAROL.—(Congres de Chirugie—Revue de Chirugie No. 5, 1893, p. 407.)

On the pathology of abscess of the liver.

The author undertook a series of experiments on the cat to elucidate this question.

The injection of dysenteric fæcal matter containing living amœbæ into the intestines of twelve animals gave in six cases abscess of the liver containing streptococci. Eleven cats contracted dysentery, seven of them having amœbæ in in the evacuations. In the intestine were found ulcerations characteristic of dysentery. In no case did he find amœbæ in sections of the intestine, but in eleven cases streptococci were found in the depths of the mucous membrane.

The injection of sterile hepatic pus into the rectum of seven cats produced in two cases abscess of the liver, in three cases a purulent infiltration, and in four cases dysentery. A pure injection of streptococci, obtained from the same source, injected into the intestine of two cats caused in one a dysentery which was cured, in the other a dysentery which caused death.

The autopsy showed ulcerations in the large intestine, and streptococci in the lungs. He argues from these experiments on animals that dysentery and abscess of the liver are of the same nature, and that the pathogenic factor is a micro-organism. Clinical observation goes to prove the same thing. The blood of three animals which were operated on for abscess of the liver, taken at the moment of operation, gave a pure culture of streptococci. Histological examination of nine cases with abscess of the liver showed streptococci in eight, and in one case staphylococci both in the pus and in sections of the liver.

The conclusions he draws are the following:

1st. Abscesses of the liver are of microbic origin.

2nd. The principal cause is the streptococcus.

3rd. Dysentery is of the same nature as abscess of the liver; and as to the amœbæ which one meets in the stools of healthy persons, they play no role in the affection.

4th. The entry of pathogenic organisms into the liver occurs most often from the intestinal canal, thence they pass to the liver either by the portal vein or by the general circulation.

